# Dried Fruit of the *Luffa* Sponge as a Source of Chitin for Applications as Skin Substitutes

**DOI:** 10.1155/2014/458287

**Published:** 2014-04-09

**Authors:** Ping-Lun Jiang, Mei-Yin Chien, Ming-Thau Sheu, Yi-You Huang, Meng-Hsun Chen, Ching-Hua Su, Der-Zen Liu

**Affiliations:** ^1^Institute of Biomedical Engineering, College of Engineering and College of Medicine, National Taiwan University, Taipei 100, Taiwan; ^2^School of Dentistry, College of Oral Medicine, Taipei Medical University, Taipei 110, Taiwan; ^3^Ko Da Pharmaceutical Co., Taoyuan 324, Taiwan; ^4^School of Pharmacy, College of Pharmacy, Taipei Medical University, Taipei 110, Taiwan; ^5^Clinical Research Center and Traditional Herbal Medicine Research Center, Taipei Medical University Hospital, Taipei 110, Taiwan; ^6^Graduate Institute of Biomedical Materials and Tissue Engineering, College of Oral Medicine, Taipei Medical University, Taipei 110, Taiwan; ^7^Department of Microbiology and Immunology, School of Medicine, College of Medicine, Taipei Medical University, Taipei 110, Taiwan; ^8^Center for General Education, Hsuan Chuang University, Hsinchu 300, Taiwan

## Abstract

*LUFFACHITIN* obtained from the residue of the sponge-like dried fruit of *Luffa aegyptiaca* was developed as a weavable skin substitute in this study. A chemical analysis revealed that *LUFFACHITIN* was composed of a copolymer containing N-acetyl-glucosamine (~40%) as a major monomer with a filamentary structure as demonstrated by both optical and scanning electron microscopy. The pulp-like white residue of the sponge-like dried fruit of *Luffa aegyptiaca* after treatment was then woven into a thin, porous membrane by filtration and lyophilization as a skin substitute for conducting wound-healing study on rats. The results indicated that the *LUFFACHITIN* membrane showed significant wound-healing enhancement (25 days to complete healing) compared to cotton gauze (>30 days), but not inferior to that of *SACCHACHITIN*. Furthermore, the *LUFFACHITIN* membrane had advantages of having a high yield, better physical properties for fabrication, and a more attractive appearance.

## 1. Introduction

In an endeavor to develop ideal skin substitutes, the performance of* SACCHACHITIN* membranes, prepared from the residue of the fruiting body of the medicinal fungus,* Ganoderma tsugae*, as an effective skin prosthesis was examined [1]. The effectiveness of the* SACCHACHITIN* membrane in managing excised wounds in guinea pigs was demonstrated to be better than that of gauze and comparable to that of Beschitin, which is mainly composed of chitin from crabs. The* SACCHACHITIN* membrane is able to promote wound healing by inducing cell proliferation. A mildly acute inflammatory reaction attracted a large number of polymorphonuclear leukocytes and some macrophages to clean away debris and blood clots [2]. Also the secretion of cell cytokines and growth factors by these cells provided an excellent environment for wound healing [3, 4]. The migration of fibroblast cells, which was promoted by* SACCHACHITIN*, also plays another important role in accelerating wound healing [1]. Furthermore, the fibrous structure of* SACCHACHITIN* made it convenient to produce a skin substitute with desirable pore characteristics.

*Luffa* is a genus of tropical and subtropical vines classified in the cucumber (Cucurbitaceae) family. In everyday nontechnical usage, the name, also spelled loofah, usually refers to the fruit of the two species,* L. aegyptiaca/cylindrical* (Smooth* Luffa*, its fruit somewhat resembles a cucumber) and* L. acutangula* (Angled* Luffa*, its fruit slightly resembles a cucumber or zucchini with ridges). The fruits of these species are cultivated and eaten as a vegetable. When the fruit is fully ripened, it is very fibrous. The fully developed fruit is the source of the loofah scrubbing sponge which is used in bathrooms and kitchens as a sponge tool.* Luffa* has fruits possessing a net-like fibrous vascular system (*Luffa* sponges) consisting of cellulose and lignin (1.4% and 2.9%, respectively, of the sponge dry weight) [5]. The struts of this natural sponge are characterized by a microcellular architecture with continuous hollow microchannels (with diameters of 10~20 *μ*m) which form vascular bundles and yield a multimodal hierarchical pore structure. To the present,* Luffa* sponges have been applied to immobilize biocatalysts such as enzymes, microorganisms, organelles, and plant and animal cells in bioreactors [6–15], scaffolds for tissue engineering [16, 17], and dye absorbents from aqueous solutions [18–20] and for developing biofiber-reinforced bionanocomposites [21–23].

The sponge vegetable from* L. aegyptiaca* is the dried fruit fiber of the sponge cucumber or sponge gourd, which is a commonly eaten vegetable in Taiwan. Similarly, the sponge-like structure of dried fruit fibers makes it suitable for cleaning the body and dishes. These dried fibers are tenacious and can be cooked for a long time with no sign of dissolution, which is similar to the characteristics of* SACCHACHITIN*. It was suspected that the main component in these fibers was chitin just as in* SACCHACHITIN*. The potential of this fibrous material for applications as a skin substitute encouraged us to conduct this study.

## 2. Materials and Methods

### 2.1. Materials

Dried fruit fibers of* L. aegyptiaca* were purchased from a local market in Taipei, Taiwan. N-acetylglucosamine, glucosamine, and ketamine were supplied by Sigma (St. Louis, MO, USA). Glucose and galactose were obtained from Nihon Shiyaku Industrial (Taipei, Taiwan). Trifluoroacetic acid and pyridine were provided by Riedel-de Haën (Seelze, Germany). n-Butanol was from Hayashi Pure Chemical (Osaka, Japan). Thin-layer chromatography (TLC) plates (Kieselgel 5554) and the solvents for high-performance liquid chromatography (HPLC) and analytical-grade reagents were obtained from Merck (Hohenbrunn, Germany). Pentobarbital was supplied by Siegfried AG (Zofingen, Switzerland).

### 2.2. Preparation of* Luffa* Membranes

The dried fruit fibers were pulverized and autoclaved for 20 min to soften the fibrous structure and then were blended to make a paste. The paste was then digested with 1 N NaOH at 85°C for 4 h. The residue was collected and washed with deionized water to remove any residual NaOH. Hypochlorite at 0.1% was then used to depigment the fibers. After removal of any residual hypochlorite by repeated washing with deionized water, the fibers of lengths ranging 10~50 *μ*m were collected and dispersed in deionized water to form a suspension. The suspension was filtered using filter paper under aseptic conditions. The membrane formed on the filter paper was then freeze-dried (EYELA, model FD-5N, Japan) to obtain the final product for further analysis and animal tests. The membrane so obtained was called “*LUFFACHITIN*.”

### 2.3. Chemical Analysis of Membrane Components

#### 2.3.1. Sugar Components [24]

Two grams of the* LUFFACHITIN* membrane was first pulverized, and then 5 mg of this powder, selected randomly, was digested with 0.25 mL of either 2 N HCl (HC-) or CF_3_COOH (HF-) at 100°C for 5 (HC-5; HF-5), 10 (HC-10; HF-10), and 15 h (HC-15; HF-15) in a sealed ampoule. After hydrolysis, each 250 *μ*L of distilled water and pyridine was added to each ampoule and mixed thoroughly. The hydrolyte residue of this mixture was divided into two portions and separately analyzed by TLC for aldose/ketose and amino sugar using glucose (galactose) and N-acetylglucosamine (glucosamine), respectively, as reference standards. The developing agent used in the TLC analysis was pyridine : n-butanol : 0.1 N HCl at 30 : 50 : 20, and the respective visualizing agents were naphthoresorcinol for aldose (purple)/ketose (red or pink) and the Elson-Morgan solution for amino sugar.

HPLC was also used to determine the kind of monosaccharides (glucose, galactose, mannose, glucosamine, and N-acetylglucosamine) in the hydrolyte of the* LUFFACHITIN* membrane. The HPLC system consisted of a pump (Shimadzu LC-10AT, Tokyo, Japan), a manual injector (Rheodyne, Tokyo, Japan), and a column (CHO-620, 250 × 4.6 mm, 5 *μ*m, Merck) operated at 63°C. The mobile phase consisted of only deionized water at a flow rate of 0.5 mL/min. The eluent was detected with an RI detector (Shimadzu, RID-10A, Tokyo, Japan).

### 2.4. Sugar Skeleton Analysis

The sugar skeleton of* LUFFACHITIN* was determined by a gas chromatography/mass spectroscopic (GC/MS) method (Hewlett Packard 5890 plus series II and Hewlett Packard, mass spectrometer 5989B). After methylation, hydrolysis, reduction, and acetylation, the pattern of fragmentation of the mass spectrum of the final product was compared to reference standards for elucidation of the sugar skeleton. GC was equipped with a capillary column (with a length of 50 m and an inner diameter of 0.25 mm) packed with dimethyl siloxane. The temperature of the injector port was 250°C, and heating was programmed from 150 to 240°C at a rate of 2°C/min. Helium was the carrier gas delivered at 1.8 mL/min with a split ratio giving a flow rate of 1.53 mL/min and maintaining a constant pressure of 1.8 kg/cm^2^. Glucose, galactose, mannose, glucosamine, and N-acetylglucosamine were used as reference standards in both the TLC and GC analyses.

### 2.5. Scanning Electronic Microscopic (SEM) Examinations

Dried samples were loaded onto aluminum studs and coated with gold for 3 min at 8 mA under a pressure of 0.1 torr. Samples were scanned and examined using a Hitachi model S-2400 SEM.

### 2.6. Wound-Healing Studies

This animal experiment was approved by the Institutional Animal Care and Use Committee of Taipei Medical University (approval number LAC-100-0101). Prior to the study, rats were anesthetized with separate intraperitoneal (i.p.) injections of ketamine (35 mg/kg) and pentobarbital (12 mg/kg). The dorsal hair of the rats was removed with an electric razor. Equal areas of two parts in a mirror image were marked along the spinal cord, 4.5 cm behind the ear of the rats and 1 cm away from the spinal cord, and two pieces of full-thickness skin, each with a surface area of about 4.0 cm^2^, were excised. The lesion on the left-hand side was covered with an equal size of* SACCHACHITIN* or cotton gauze for comparison. The right-hand side was covered with the* LUFFACHITIN* membrane prepared above. Treated rats were placed in individual cages with an air-filtering device at 30°C and 55±% humidity, where they had free access to food and water. After surgery, changes in the area of the wounds were measured on days 4, 7, 11, 14, 18, 21, and 25, after which fresh dressings were applied. A digital camera was used to document the lesion, and Image-Pro Plus was used to calculate the wound area on the image of the lesion so obtained. Five male rats for each group were included in two comparative studies, and results are reported as the mean with the standard deviation (SD). Student's *t*-test was performed using the SPSS statistic software (PASW Statistics 18.0). Difference was considered significant when the *P* value was less than 0.05.

## 3. Results

**Figure 1:**
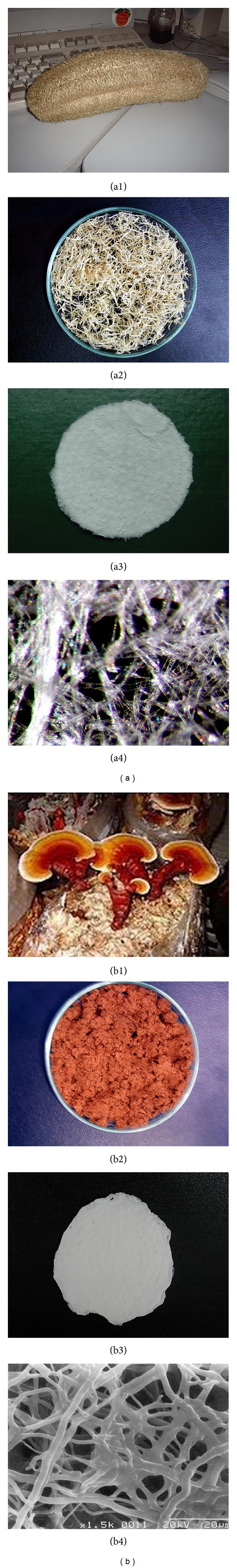
Appearance of the* LUFFACHITIN* membrane collected at different stages of purification. (a)* Luffa aegyptiaca* and (b)* Ganoderma*; (1) original material; (2) residue after alkaline treatment; (3) the final product; (4) microscopic photographs. The scale bar of (a4) is the same with that of (b4).

Figure 1 shows the appearance of* LUFFACHITIN* (Figure 1(a1), from* L. aegyptiaca*) developed in different stages of treatment compared to that of* SACCHACHITIN* (Figure 1(b1), from the fruiting body of* Ganoderma*). Dried fruit fibers of* L. aegyptiaca* appeared fiber-like with a light-yellow color as shown in Figures 1(a2) and 1(a3), whereas the fruiting body of* Ganoderma* was a reddish-brown color with a less-fibrous form and a more-tendon-like structure demonstrated by Figures 1(b2) and 1(b3). Microscopic examination of the* LUFFACHITIN* membrane (Figure 1(a4)) clearly showed that the light-yellow fibers of* L. aegyptiaca* were transformed into soft, transparent fibers that formed a porous structure, while* SACCHACHITIN* fibers appeared to be opaque that formed a porous structure as well (Figure 1(b4)).

**Figure 2:**
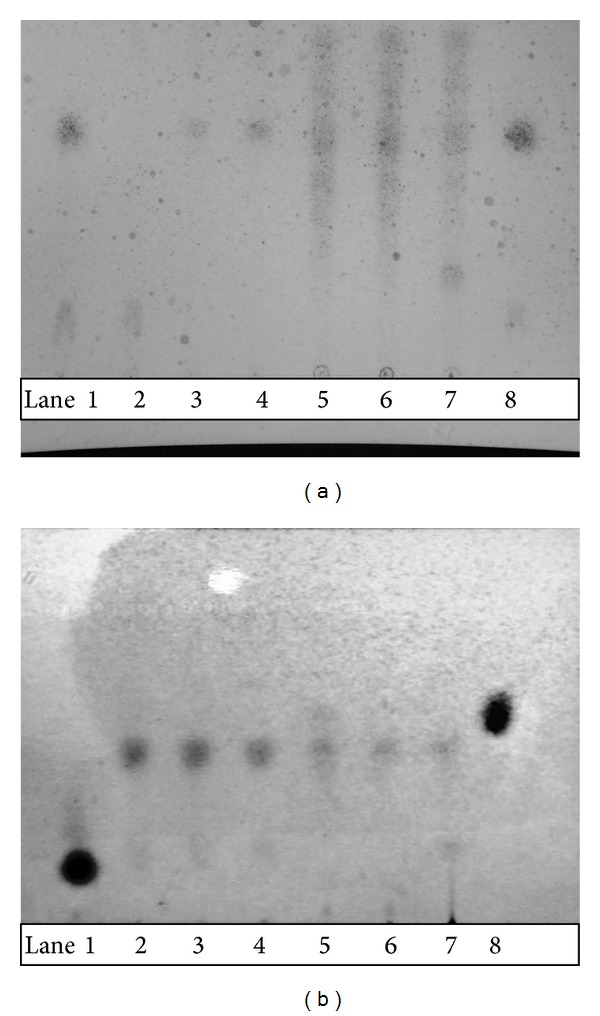
Thin-layer chromatography of the acid-treated hydrolysate (HCl: HC-5, HC-10, and HC-15; CF_3_COOH: HF-5, HF-10, and HF-15) of the* LUFFACHITIN* membrane developed by pyridine : n-butanol : 0.1 N HCl at 30 : 50 : 20 and visualized by spraying the naphthoresorcinol reagent (a) or Elson-Morgan reagent (b) and heating to 100°C for 3 min. Lanes 1~8: glucose (N-acetylglucosamine), HC-5, HC-10, HC-15, HF-5, HF-10, HF-15, and galactose (glucosamine).

The TLC analysis of the acid-treated hydrolyte of* LUFFACHITIN* membrane is shown in Figure 2. With hydrolysis at the same concentration and time interval, a spot visualized by naphthoresorcinol to be purple appeared in all hydrolytes except that reacting with HCl for only 5 h (HCl-5, Figure 2(a)) as shown in Figure 2(a). Since a purple spot was visualized after treatment with naphthoresorcinol and appeared at similar retention time with glucose/galactose standard in TLC plate, it was concluded that aldose of glucose/galactose is one of the major sugar components of* LUFFACHITIN*.  Figure 2(b) reveals the presence of N-acetylglucosamine in all acid-treated hydrolytes of the* LUFFACHITIN* membrane, which was confirmed by referring to the N-acetylglucosamine standard with visualization of a purple-violet color. Another purple spot corresponding to the glucosamine standard only appeared in hydrolytes treated with a stronger acid for the longest time interval (CF-15, lane 8, Figure 2(b)). It was concluded that amino sugar of N-acetylglucosamine is another major sugar component of* LUFFACHITIN*.

**Figure 3:**
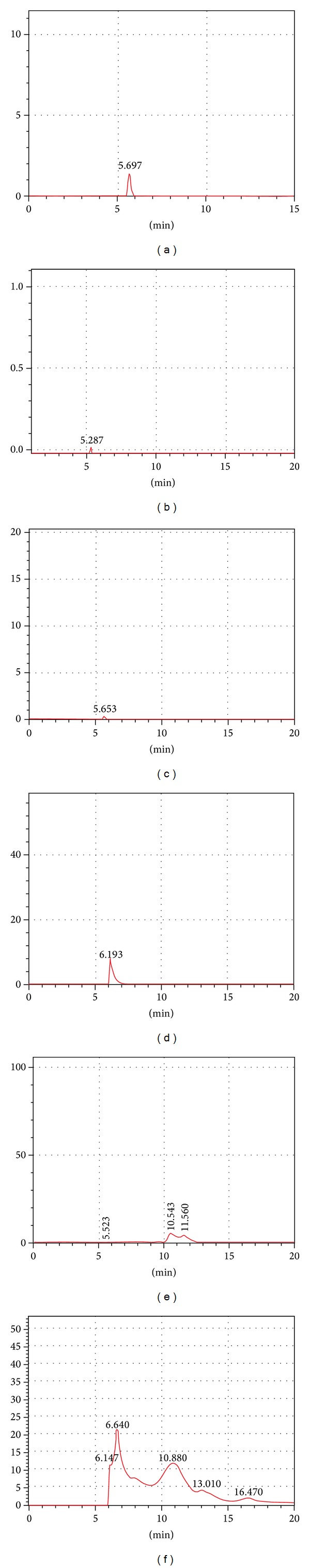
HPLC analytical graphs for various sugar standards and HF-15. (a) Glucose (5 mg/mL, 5.697 min); (b) galactose (1 mg/mL, 5.287 min); (c) mannose (1 mg/mL, 5.653 min); (d) glucosamine (0.5 mg/mL, 6.193 min); (e) N-acetyl-D-glucosamine (0.5 mg/mL, minor/5.523; major/10.543 and 11.560 min); (f) HF-15.

Figures 3(a)–3(e) illustrated the results of HPLC analysis for monosaccharide standards of glucose, galactose, mannose, glucosamine, and N-acetylglucosamine, respectively, and Figure 3(f) revealed that for acidic hydrolytes of* LUFFACHITIN* as exemplified by HF-15, which was hydrolyzed in CF_3_COOH for 15 h. In comparison with aldose standards, it is obvious that the major broad peak in HPLC graph of HF-15 at a retention time around 5.5 min corresponds with the mixture of aldoses, including glucose (5.697 min), galactose (5.287 min), and mannose (5.653 min). This result conforms to that of TLC as deduced above. Another major peak in HPLC graph of HF-15 appears at a retention time similar to that of N-acetylglucosamine standard (Figure 3(e)) but not glucosamine (Figure 3(d)). This result is also consistent with that revealed by TLC analysis. Since a complete acidic hydrolysis of* LUFFACHITIN* in CF_3_COOH for 15 h was expectable, the area under the peak corresponding to N-acetylglucosamine would be the highest amount of N-acetylglucosamine released by acidic treatment of* LUFFACHITIN*. The area under this peak was calculated to be ~40% of total peak area in HPLC graph, which means that* LUFFACHITIN* is composed of ~40% N-acetylglucosamine and the rest of 60% is glucose (or a mixture of aldoses).

**Figure 4:**
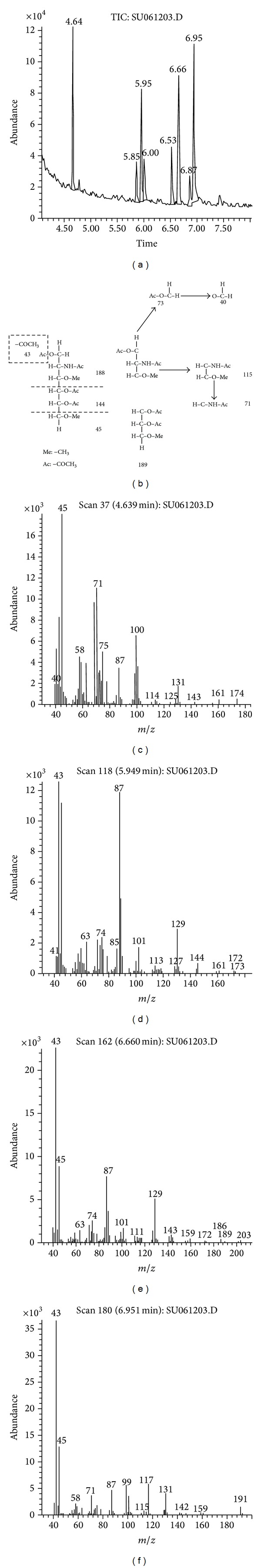
Gas chromatography/mass spectrum (GC/MS) of the final product of* LUFFACHITIN* after methylation, hydrolysis, reduction, and acetylation treatments. (a) GC chromatography; (b) proposed fragmentation of poly-N-acetylglucosamine after treatment; (c)–(f) mass spectra of respective elution peaks at 4.64, 5.95, 6.66, and 6.95 min.

Figure 4 shows a GC graph (Figure 4(a)) of the final products of* LUFFACHITIN* after methylation, hydrolysis, reduction, and acetylation treatments with corresponding mass spectra for elution peaks at 4.64 (Figure 4(c)), 5.95 (Figure 4(d)), 6.66 (Figure 4(e)), and 6.95 (Figure 4(f)) min.  Figure 4(b) reveals that the characteristic fragments for N-acetylglucosamine in the structure of poly[*β*-1,4-N-acetyl-D-glucosamine] included 45, 71, 73, and 189 m/z. The mass spectrum of the elution peak at 6.66 min demonstrated those characteristic fragments, further confirming poly[*β*-1,4-N-acetyl-D-glucosamine] to be part of the structural unit of* LUFFACHITIN*. Following the same principle as elucidated by the fragmentation of poly[*β*-1,4-N-acetyl-D-glucosamine], characteristic fragments for poly-*β*-1,4-glucose and poly-*β*-1,3-glucose (*β*-1,3-glucan) were 45, 117, 161, and 205 m/z and 45, 117, 161, and 233 m/z, respectively. All those characteristic fragments except 205 m/z for poly-*β*-1,4-glucose and 233 m/z for poly-*β*-1,3-glucose appeared in the mass spectra for elution peaks at 4.64, 5.95, and 6.95 min. It seems to indicate that besides the most abundant structural units of poly-*β*-1,4-glucose in nature being a part of structural unit of* LUFFACHITIN*, poly-*β*-1,3-glucose backbone could not be excluded.

**Figure 5:**
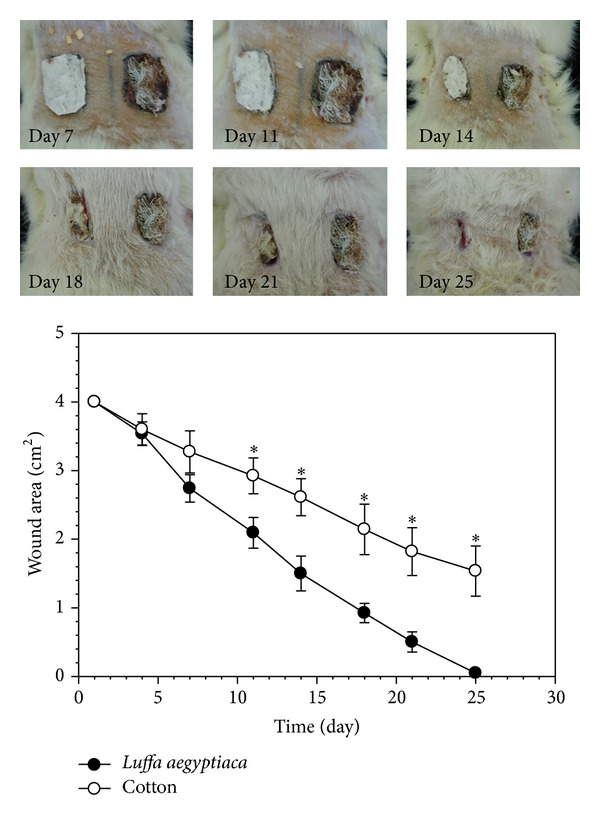
Top panel: example of photographic illustrations of the wound-healing process using covering with either cotton gauze (right-hand side) or* LUFFACHITIN* membrane (left-hand side) on days 7, 11, 14, 18, 21, and 25. Bottom panel: comparison of the contraction curves of the wound areas after treatment with cotton gauze (○) and the* LUFFACHITIN* membrane (●) (*n* = 5). *P < 0.05.

**Figure 6:**
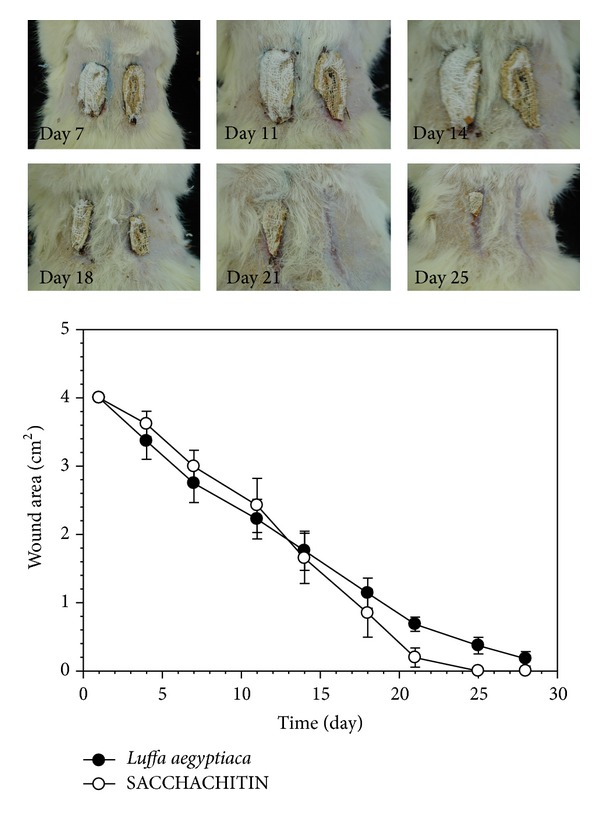
Top panel: example of photographic illustrations of the wound-healing process covered with either a* SACCHACHITIN* (right-hand side) or* LUFFACHITIN* membrane (left-hand side) on days 7, 11, 14, 18, 21, and 25. Bottom panel: comparison of the contraction curves of the wound areas after treatment with the* SACCHACHITIN* (○) and* LUFFACHITIN* membranes (●) (*n* = 5). *P < 0.05.

The wound-healing process for the wound covered by the* LUFFACHITIN* membrane was compared to both cotton gauze and* SACCHACHITIN*. Photographic results are shown in the upper panel of Figures 5 and 6. The wound area contraction plotted versus time was shown in the bottom panels of Figures 5 and 6, respectively, to compare the progress of wound healing using these different skin substitutes or wound dressings. In comparison to the control covered with cotton gauze, quite significant improvement in the wound-healing process with the* LUFFACHITIN* membrane was observed by the change in the wound area. The difference between the* LUFFACHITIN* and* SACCHACHITIN* membranes was observed to be minimal. The wound lesion of 4.0-cm^2^ had completely healed after covering with either substance for 25 days. Skin tissue with normal function recovered, and dorsal hairs also regrew in the healed wound area.

## 4. Discussion

During treatment, it was found that a longer time in harsher conditions was taken to soften and depigment the* SACCHACHITIN* preparation than the* LUFFACHITIN* preparation. Because of that, the total recovery for* LUFFACHITIN* was about 70%, whereas it was only about 20% for* SACCHACHITIN*. To be effective as a skin substitute or dressing to treat wounds like with* SACCHACHITIN*, the porous structures that appeared in the* LUFFACHITIN* membrane were recognized as an important characteristic. Microscopic examination clearly showed that the* LUFFACHITIN* membrane is composed of soft and transparent fibers that formed a porous structure. The pore size of these membranes could be optimally controllable by adjusting the fiber concentration of the filtrate during preparation of the membrane. Furthermore, the* LUFFACHITIN* membrane was softer like facial tissues with better water-absorption ability than the* SACCHACHITIN* membrane (personal observation). The softness allowed the membrane to easily attach to the skin contour, and the better ability to absorb water led to the efficient expulsion of exudates. It would be expectable to have a minimal injury to skin surface where it attached to and enhance the skin recovery.

It was confirmed that the main constituents of* LUFFACHITIN* are aldose of glucose/galactose and amino sugar of N-acetylglucosamine by TLC and HPLC. The acetylation products of the hydrolyte determined by GC analysis (data not shown) were also demonstrated to be these two ingredients (glucose and N-acetylglucosamine). In comparison with a calibration standard curve, the ratio of these two components was found to be 60.15 to 39.85. The TLC diagram was also used to validate that the sugar components are released by digestion with various enzymes (data not shown). It demonstrates that only *β*-glucosidase and chitinase are able to hydrolyze* LUFFACHITIN* and the resulting sugar components were determined to be glucose and N-acetylglucosamine. Measurement of the protein content of* LUFFACHITIN* produced no color reaction implying that* LUFFACHITIN* contains virtually no protein. The total nitrogen content was determined to be 3.46%, which was equivalent to the total nitrogen content of N-acetylglucosamine, further confirming that sponge gourd from* Luffa aegyptiaca* is composed mainly of the *β*-form of glucan and poly-N-acetylglucosamine.

Regarding the wound-healing improvement, quite significant improvement with the* LUFFACHITIN* membrane was observed in comparison to the control covered with cotton gauze. The difference between the* LUFFACHITIN* and* SACCHACHITIN* membranes was observed to be minimal. Overall, the* LUFFACHITIN* membrane could be utilized as a wound dressing or skin substitute with an efficacy comparable to that of* SACCHACHITIN* but better than gauge. As reposted, the chitin (polymeric N-acetyl-glucosamine) was demonstrated to play an important role for chitin-containing materials in the wound-healing process [25–27]. Previously, the proliferation of human F1000 fibroblasts was also found to correlate with the chitin content of various fungal cultures [28]. As revealed above, N-acetylglucosamine is one of the two main components of the* LUFFACHITIN* membrane. Therefore, the comparable efficacy of the* LUFFACHITIN* membrane with respect to* SACCHACHITIN* in the wound-healing process can partly be attributed to N-acetylglucosamine, a major monomer in the structure of chitin. In addition, the immunological effects and influence of *β*-1,3-glucan on wound healing are elucidated in greater detail elsewhere [29–31]. The potential possibility of a 1,3-linkage for polysaccharides obtained from* L. aegyptiaca* is illustrated although the linkage of the glucose unit has not been confirmed. Since being another main component in the* LUFFACHITIN* membrane, the potential role of *β*-1,3-glucan in the promotion of wound healing and in the retardation of scar formation cannot be ignored. Biochemical evaluations of the wound-healing process treated with the* LUFFACHITIN* membrane are under progress.

## References

[1] Su C-H, Sun C-S, Juan S-W, Hu C-H, Ke W-T, Sheu M-T (1997). Fungal mycelia as the source of chitin and polysaccharides and their applications as skin substitutes. *Biomaterials*.

[2] Su C-H, Liu S-H, Yu S-Y (2005). Development of fungal mycelia as a skin substitute: characterization of keratinocyte proliferation and matrix metalloproteinase expression during improvement in the wound-healing process. *Journal of Biomedical Materials Research Part A*.

[3] Gill SE, Parks WC (2008). Metalloproteinases and their inhibitors: regulators of wound healing. *International Journal of Biochemistry and Cell Biology*.

[4] Chittoria RK (2012). Role of growth factors in wound healing. *Journal of Society For Wound Care and Research*.

[5] Chang S-C, Lee M-S, Li C-H (1995). Dietary fibre content and composition of vegetables in Taiwan area. *Asia Pacific Journal of Clinical Nutrition*.

[6] Pazzetto R, Ferreira SB, Santos EJ (2012). Preservation of Bacillus firmus strain 37 and optimization of cyclodextrin biosynthesis by cells immobilized on loofa sponge. *Molecules*.

[7] Meleigy SA, Khalaf MA (2009). Biosynthesis of gibberellic acid from milk permeate in repeated batch operation by a mutant Fusarium moniliforme cells immobilized on loofa sponge. *Bioresource Technology*.

[8] Kar S, Swain MR, Ray RC (2009). Statistical optimization of alpha-amylase production with immobilized cells of Streptomyces erumpens MTCC 7317 in *Luffa cylindrica* l. sponge discs. *Applied Biochemistry and Biotechnology*.

[9] Saudagar PS, Shaligram NS, Singhal RS (2008). Immobilization of Streptomyces clavuligerus on loofah sponge for the production of clavulanic acid. *Bioresource Technology*.

[10] Phisalaphong M, Budiraharjo R, Bangrak P, Mongkolkajit J, Limtong S (2007). Alginate-loofa as carrier matrix for ethanol production. *Journal of Bioscience and Bioengineering*.

[11] Hideno A, Ogbonna JC, Aoyagi H, Tanaka H (2007). Acetylation of loofa (*Luffa cylindrica*) sponge as immobilization carrier for bioprocesses involving cellulase. *Journal of Bioscience and Bioengineering*.

[12] Ganguly R, Dwivedi P, Singh RP (2007). Production of lactic acid with loofa sponge immobilized Rhizopus oryzae RBU2-10. *Bioresource Technology*.

[13] Iqbal M, Saeed A, Edyvean RGJ, O’Sullivan B, Styring P (2005). Production of fungal biomass immobilized loofa sponge (FBILS)-discs for the removal of heavy metal ions and chlorinated compounds from aqueous solution. *Biotechnology Letters*.

[14] Iqbal M, Zafar SI (1994). Vegetable sponge as a matrix to immobilize micro-organisms: a trial study for hyphal fungi, yeast and bacteria. *Letters in Applied Microbiology*.

[15] Pekdemir T, Keskinler B, Yildiz E, Akay G (2003). Process intensification in wastewater treatment: ferrous iron removal by a sustainable membrane bioreactor system. *Journal of Chemical Technology and Biotechnology*.

[16] Chen J-P, Lin T-C (2005). Loofa sponge as a scaffold for culture of rat hepatocytes. *Biotechnology Progress*.

[17] Chen J-P, Yu S-C, Hsu BR-S, Fu S-H, Liu H-S (2003). Loofa sponge as a scaffold for the culture of human hepatocyte cell line. *Biotechnology Progress*.

[18] Altinişik A, Gür E, Seki Y (2010). A natural sorbent, *Luffa cylindrica* for the removal of a model basic dye. *Journal of Hazardous Materials*.

[19] El Ashtoukhy ESZ (2009). Loofa egyptiaca as a novel adsorbent for removal of direct blue dye from aqueous solution. *Journal of Environmental Management*.

[20] Demir H, Top A, Balköse D, Ülkü S (2008). Dye adsorption behavior of *Luffa cylindrica* fibers. *Journal of Hazardous Materials*.

[21] Siqueira G, Bras J, Follain N (2013). Thermal and mechanical properties of bio-nanocomposites reinforced by *Luffa cylindrica* cellulose nanocrystals. *Carbohydrate Polymers*.

[22] Boynard CA, Monteiro SN, D’Almeida JRM (2003). Aspects of alkali treatment of sponge gourd (*Luffa cylindrica*) fibers on the flexural properties of polyester matrix composites. *Journal of Applied Polymer Science*.

[23] Siqueira G, Bras J, Dufresne A (2010). *Luffa cylindrica* as a lignocellulosic source of fiber, microfibrillated cellulose, and cellulose nanocrystals. *BioResources*.

[24] Chaplin CF, Chaplin MF, Kennedy JF (1987). Monosaccharides. *Carbohydrate Analysis*.

[25] Balassa LL Use of chitin for promoting wound healing.

[26] Balassa LL Chitin and chitin derivatives for promoting wound healing.

[27] Singh R, Singh D (2012). Chitin membranes containing silver nanoparticles for wound dressing application. *International Wound Journal*.

[28] Lip Yong Chung LYC, Schmidt RJ, Hamlyn PF, Sagar BF, Andrews AM, Turner TD (1994). Biocompatibility of potential wound management products: fungal mycelia as a source of chitin/chitosan and their effect on the proliferation of human F1000 fibroblasts in culture. *Journal of Biomedical Materials Research*.

[29] Karaaslan O, Kankaya Y, Sungur N (2012). Case series of topical and orally administered beta-glucan for the treatment of diabetic wounds: clinical study. *Journal of Cutaneous Medicine and Surgery*.

[30] Kiho T, Sakushima M, Wang S, Nagai K, Ukai S (1991). Polysaccharides in fungi. XXVI. Two branched (1 → 3)-*β*-D-glucans from hot water extract of Yuĕr. *Chemical and Pharmaceutical Bulletin*.

[31] Saito K, Nishijima M, Miyazaki T (1990). Further examination on the structure of an alkali-soluble glucan isolated from Omphalia lapidescens. Studies on fungal polysaccharide. XXXVI. *Chemical and Pharmaceutical Bulletin*.

